# Supplementary material for: Predicting visual function by interpreting a neuronal wiring diagram
Source: Nature. 2024 Oct 2;634(8032):113–23. doi: 10.1038/s41586-024-07953-5 (PMC11446822; doi:10.1038/s41586-024-07953-5)
Supplement: Supplementary file 6 — CRF and ERF predictions for individual TmY4 and TmY9 cells. Analogous to Supplementary Data 3, but for TmY target types. Shown are the top four monosynaptic pathways, the strongest pathway passing through each of the top ten intermediary types (ranking from Extended Data Fig. 7), and the trisynaptic pathway Tm1–TmY–Dm3–TmY (see the section entitled Prediction of spatial normalization). [file 41586_2024_7953_MOESM6_ESM.zip › DataS4/TmY9q/720575940638682601.pdf]

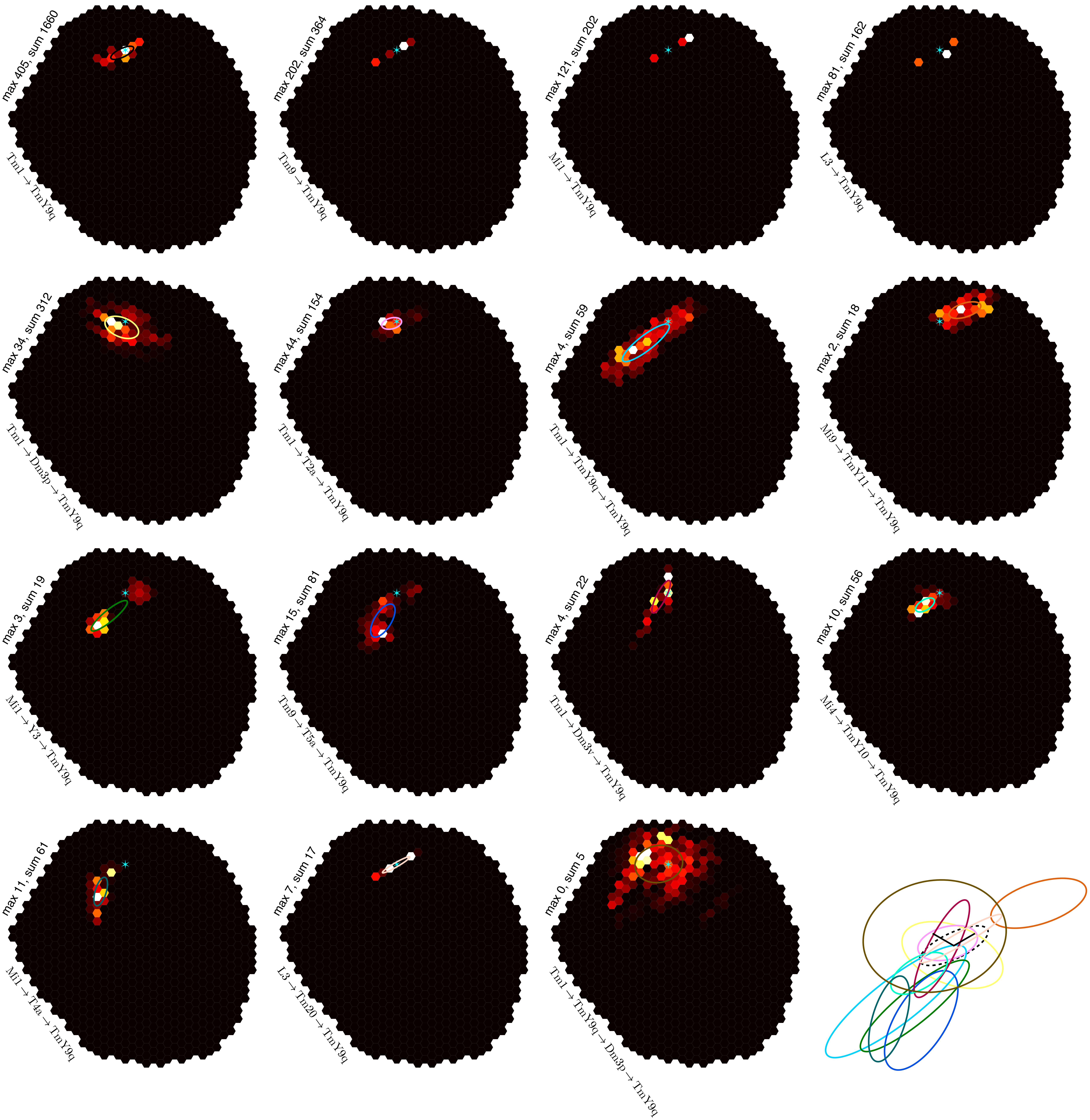
max 405, sum 1660
Tm1 → TmY9q
max 202, sum 364
Tm9 → TmY9q
max 121, sum 202
Mi1 → TmY9q
max 81, sum 162
L3 → TmY9q
max 34, sum 312
Tm1 → Dm3p → TmY9q
max 44, sum 154
Tm1 → T2a → TmY9q
max 4, sum 59
Tm1 → TmY9q → TmY9q
max 2, sum 18
Mi9 → TmY11 → TmY9q
max 3, sum 19
Mi1 → Y3 → TmY9q
max 15, sum 81
Tm9 → T5a → TmY9q
max 4, sum 22
Tm1 → Dm3v → TmY9q
max 10, sum 56
Mi4 → TmY10 → TmY9q
max 11, sum 61
Mi1 → T4a → TmY9q
max 7, sum 17
L3 → Tm20 → TmY9q
max 0, sum 5
Tm1 → TmY9q → Dm3p → TmY9q
